# Spontaneous mutations in the *flhD* operon generate motility heterogeneity in *Escherichia coli* biofilm

**DOI:** 10.1186/s12866-016-0878-1

**Published:** 2016-11-08

**Authors:** Shelley M. Horne, Joseph Sayler, Nicholas Scarberry, Meredith Schroeder, Ty Lynnes, Birgit M. Prüß

**Affiliations:** Department of Veterinary and Microbiological Sciences, North Dakota State University, Fargo, ND 58103 USA

**Keywords:** Motility heterogeneity, Biofilm, *Escherichia coli* K-12, Mutations, IS elements

## Abstract

**Background:**

Heterogeneity and niche adaptation in bacterial biofilm involve changes to the genetic makeup of the bacteria and gene expression control. We hypothesized that i) spontaneous mutations in the *flhD* operon can either increase or decrease motility and that ii) the resulting motility heterogeneity in the biofilm might lead to a long-term increase in biofilm biomass.

**Results:**

We allowed the highly motile *E. coli* K-12 strain MC1000 to form seven- and fourteen-day old biofilm, from which we recovered reduced motility isolates at a substantially greater frequency (5.4 %) than from a similar experiment with planktonic bacteria (0.1 %). Biofilms formed exclusively by MC1000 degraded after 2 weeks. In contrast, biofilms initiated with a 1:1 ratio of MC1000 and its isogenic *flhD*::*kn* mutant remained intact at 4 weeks and the two strains remained in equilibrium for at least two weeks. These data imply that an ‘optimal’ biofilm may contain a mixture of motile and non-motile bacteria.

Twenty-eight of the non-motile MC1000 isolates contained an IS1 element in proximity to the translational start of FlhD or within the open reading frames for FlhD or FlhC. Two isolates had an IS2 and one isolate had an IS5 in the open reading frame for FlhD. An additional three isolates contained deletions that included the RNA polymerase binding site, five isolates contained point mutations and small deletions in the open reading frame for FlhC. The locations of all these mutations are consistent with the lack of motility and further downstream within the *flhD* operon than previously published IS elements that increased motility. We believe that the location of the mutation within the *flhD* operon determines whether the effect on motility is positive or negative.

To test the second part of our hypothesis where motility heterogeneity in a biofilm may lead to a long-term increase in biofilm biomass, we quantified biofilm biomass by MC1000, MC1000 *flhD*::*kn*, and mixtures of the two strains at ratios of 1:1, 10:1, and 1:10. After 3 weeks, biofilm of the mixed cultures contained up to five times more biomass than biofilm of each of the individual strains.

**Conclusion:**

Mutations in the *flhD* operon can exert positive or negative effects on motility, depending on the site of the mutation. We believe that this is a mechanism to generate motility heterogeneity within *E. coli* biofilm, which may help to maintain biofilm biomass over extended periods of time.

**Electronic supplementary material:**

The online version of this article (doi:10.1186/s12866-016-0878-1) contains supplementary material, which is available to authorized users.

## Background

Bacteria use diverse mechanisms to adapt to their environment. Adaptation at the level of gene expression is often short term; for example to survive at high [1] or low [2] temperature after a sudden shift. Selection for specific mutations in response to selective pressure can lead to antibiotic resistance (for a recent review, see [3]). This study investigates a combination of adaptive gene expression with selective mutation by individual bacteria within a biofilm. Biofilm is defined as bacterial colonies encased in a matrix that form on surfaces, at air-liquid interfaces, or within eukaryotic cells. Distinct environmental niches within the biofilm are characterized by the availability of oxygen or nutrients, as well as exposure to antibiotics and/or the immune system. While bacteria respond to these niches at an individual level, the differential responses of many bacteria can lead to phenotypic heterogeneity across the biofilm, in particular in cases where the selective pressure is insufficient to clearly favor one or the other phenotype.

Three examples of adaptive gene expression leading to motility heterogeneity in a biofilm come from *Bacillus subtilis* [4], *Escherichia coli* K-12 [5], and *Salmonella enterica* [6–8]. In *B. subtilis*, genes of motility, matrix production, and sporulation are expressed in a spatiotemporal order [4]. In the study by our own laboratory, expression of the *E. coli* flagellar master regulator FlhD_4_ FlhC_2_ (FlhD/FlhC) was limited to the outermost edge of the biofilm at specific times during biofilm development [5]. In *S. enterica*, motility bistability enabled the bacteria to perform a division of labor, where motile cells contribute to the infection and non-motile cells serve as a reservoir [6–8]. Many examples of selective mutation include a range of DNA transfer and recombination events, such as horizontal DNA transfer, conjugation, transformation, and intraspecies DNA transfer by mobilizable elements [9]. An intriguing combination of adaptive gene expression and selective mutation are IS elements, where the initial evolutionary event of the IS element insertion into genes or operons can lead to changes in gene expression, dysfunctional proteins, and phenotypic changes (for a review on mutations by transposons, see [10]).

The *flhD* operon that controls expression of the FlhD/FlhC transcriptional activator and hence the expression of all other flagellar genes is subject to IS element insertion. Barker and coworkers determined that insertion of an IS5 element into the *flhD* promoter of the originally non-motile version of *E. coli* MG1655 Fnr- resulted in a 2.7-fold increase in *flhD* expression and a 7-fold increase in the rate of migration through semi-solid agar [11]. Wang and Wood determined that an IS5 in the *flhD* promoter of BW25113 increased both motility and biofilm amounts [12]. In both these previous reports [11, 12], IS5 inserted into the same 4-bp target site (5’-TTAA-3’) located 96–99 bp upstream of the transcriptional start of *flhD,* causing a poorly motile strain to become considerably more motile. A third group led by C. Park selected motile isolates from the non-motile version of MG1655 and found IS elements even further upstream of the transcriptional start for *flhD*. Relative to the transcription start site, two IS5 elements inserted 315 and 166 bp upstream, while one IS1 inserted 303 bp upstream [13]. The final study that identified insertion elements in the *flhD* promoter is by Fahrner and Berg, who found IS elements up to 476 bp from the transcriptional start that still had stimulating effects on expression of the *flhD* operon [14]. The authors concluded that a large upstream region is involved in the topology of *flhD* expression.

The aforementioned IS elements in the *flhD* operon of different *E. coli* K-12 backgrounds have several characteristics in common: i) the mutants were obtained from genetic background strains that were non-motile prior to the experiment and ii) IS elements inserted upstream of the transcriptional start of the operon, where they increased *flhD* expression and enhanced motility.

With this study, we used the highly motile genetic background strain MC1000. We recovered non-motile isolates from biofilm formed by this strain that carried mutations in their *flhD* operon further downstream than the previously reported isolates with increased motility. We conclude that the location of the mutation within the *flhD* operon determines the effect on motility and propose this as a mechanism to generate motility heterogeneity in *E. coli* K-12 biofilm. We hypothesize that this motility heterogeneity may help the bacteria to maintain a biofilm for a longer period of time, leading to an increased biofilm biomass relative to the parental strain after prolonged incubation.

## Results

### Motility heterogeneity in *E. coli* K-12 biofilm

Bacterial isolates were recovered from biofilms produced by the highly motile MC1000 after 7 and 14 days. From the 7-day old biofilm, we recovered 368 isolates; from the 14-day old biofilm, we recovered 1217 isolates. We tested the total of 1585 isolates for motility (Table 1, motility column). From the 7-day old biofilm, we identified 4 non-motile isolates and 9 isolates with reduced motility. From the 14-day old biofilm, we recovered 58 non-motile isolates and 14 isolates with reduced motility. To determine whether the tendency towards reduced motility is a feature of biofilm formation, we tested planktonic MC1000 bacteria. In three replicate experiments, 2070 isolates were recovered from 14-day old aerated cultures; three of these isolates were non-motile. The reduced or non-motile MC1000 biofilm isolates were designated JS17 through JS101 (Table 1, colony designations column); the planktonic MC1000 isolates were designated JS107 to JS109. The greater frequency of reduced or non-motile isolates isolated from the biofilm relative to the planktonic bacteria (5.4 % versus 0.1 %) suggests that the biofilm environment of MC1000 may have selected for non-motile isolates. Note that ‘Additional file 1’ that is provided as online supplement contains raw data from experiments that were performed with isolates JS1 through JS101 at the level of the individual isolate. Table 1 of this manuscript categorizes isolates.

**Table 1:** Summary of phenotypes for isolates that were recovered from biofilm of MC1000

Incubation	# of col.	Motility^a^	Colony designations^b^	pXL27 compl.^c^	PCR1/PCR2^d^				PCR3^e^
					Partially Motile	Non-Motile			
						Category I	Category II	Category III	
7d	368	4 NM (1 %)	JS28-31	4 col. PM		4 col. with parental PCR fragments			ND
		9 PM (2.4 %)	JS19-JS27	ND	9 col. with parental PCR fragments				ND
14 d	1217	58 NM (4.8 %)	JS18, JS42-79, JS82-JS100	54 col. FM, 4 col. PM		21 col. with parental PCR fragments	3 col. lacking a PCR1 product, 3 col. lacking a PCR2 product	31 col. with extended PCR fragments in both reactions	31 col. tested: 6 col. JS44 orient, 22 col. JS90 orient, 3 col. no PCR3 fragments
		14 PM (1.1 %)	S17, JS32-JS41, JS80, JS81, JS101	ND	14 col. with parental PCR fragments				ND

^a^Motility was determined on motility plates: NM, the isolate was completely non-motile; PM, the isolate exhibited partial motility relative to its parent strain

^b^All 85 MC1000 biofilm isolates that exhibited reduced motility relative to MC1000 were given JS designations

^c^ Non-motile isolates from MC1000 biofilm were tested for complementation with pXL27: FM, at least 7 of 8 transformants exhibited the full motility of the parent; PM, fewer than 7 of 8 transformants were fully motile or all colonies were partially motile. ND, not determined

^d^Reduced motility isolates from MC1000 biofilm were subjected to PCR1 and PCR2 to test for insertions/deletions within the *flhD* operon

^e^The 31 isolates from category III were subjected to PCR3 to identify IS1 insertions

To determine whether extended times of incubation could increase the percentage of non-motile bacteria in MC1000 biofilm, we tried to recover isolates from 1-month old biofilms. In duplicate experiments, the biofilm disintegrated after 2 weeks. At 4 weeks, we recovered only between 10^4^ and 10^5^ colony forming units (CFU) from a surface area of 28.3 cm^2^ (data not shown).

**Fig. 1:**
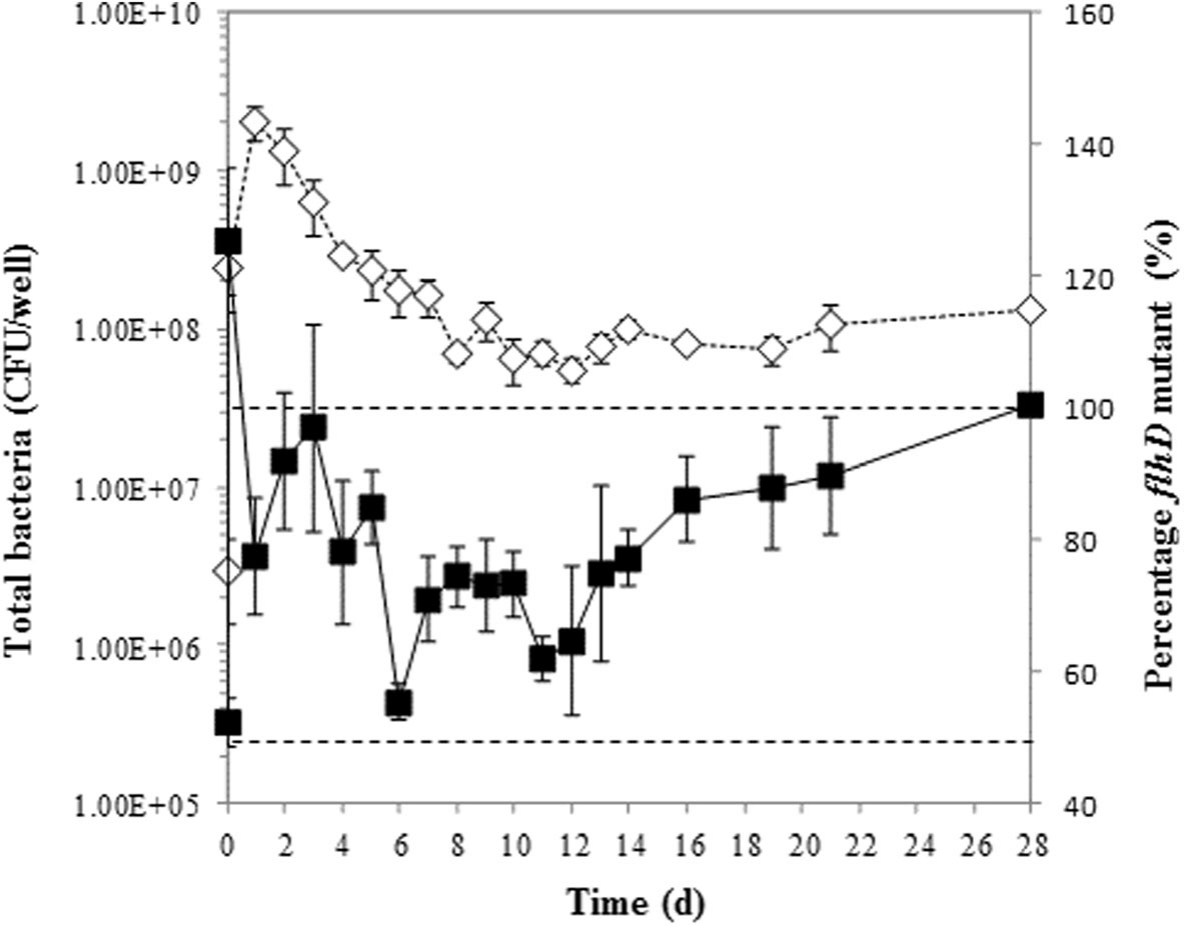
Competitive biofilm experiment. MC1000 and BP19 were grown at a starting ratio of 1:1. Biofilm bound bacteria were enumerated on LB and LB/kn plates. The total number of bacteria (*white diamonds, dashed line*) is presented as colony forming units per well (CFU/well) and was calculated from the LB plates. To determine the percentage of *flhD* mutants (black squares, solid line), bacterial counts from the kanamycin plates were divided by total bacterial numbers. Dashed lines mark 50 and 100 % *flhD* mutants

We next initiated biofilm formation with a 1:1 mixture of MC1000 and its isogenic MC1000 *flhD*::*kn* mutant (designated BP19) and monitored the number of total and mutant bacteria over the course of 4 weeks. After the first day, the maximal total number of biofilm bound bacteria was 2 × 10^9^ CFU/well on a surface area of 9.6 cm^2^ (Fig. 1, open diamonds, dashed line). Bacterial numbers gradually decreased to about10^8^ CFU/well. The percentage of *flhD* mutants started at approximately 50 %, which is consistent with the intended 1:1 inoculation of the two bacterial strains. During the first 2 weeks, mutant percentage fluctuated between 50 and 100 %; over the next 2 weeks, it increased steadily to 100 % (Fig. 1, closed squares, solid line). An initial sharp peak in the percentage of *flhD* mutants will be addressed in the Discussion. We conclude that MC1000 biofilm maintains heterogeneity for at least 2 weeks.

### Characterization of the non-motile MC1000 isolates

Non-motile isolates recovered from MC1000 biofilm were characterized further. To test for mutations in the *flhD* operon, we attempted to complement the lack of motility with the *flhD-*expressing plasmid pXL27 (Table 1, pXL27 complementation column). Of the 62 isolates tested, 54 complemented to full MC1000 motility, suggesting the presence of mutations within the *flhD* operon. The other 8 non-motile isolates complemented to partial MC1000 motility. The lack of full complementation suggests that the loss of motility resulted from a non-*flhD* mutation, perhaps in other flagella genes; these isolates were not further characterized.

**Fig. 2:**
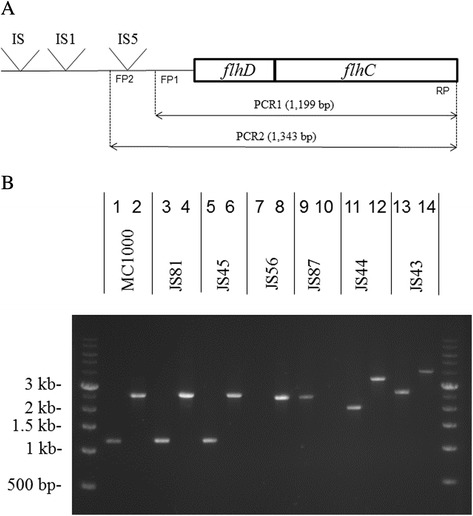
PCR1 and PCR2. **a** details the *flhD* operon and the two PCR reactions [11]. PCR1 is expected to yield a 1199 bp product, PCR2 is expected to yield a 1344 bp product in the absence of insertions or deletions. FP1stands for Forward Primer for PCR1, FP2 stands for Forward Primer for PCR2, RP stands for Reverse Primer for both PCR1 and PCR2. IS5 refers to the IS5 element at position −99 to −96 from the transcriptional start that was described by Barker *et al.* [11] and Wang and Wood [12]. IS1 refers to the IS1 elements that was described by Lee and Park [13] at −303 pb. IS refers to the IS5 described by Fahrner and Berg as the most distal (−476) IS element that had a positive effect on *flhD* expression [14]. **b** contains the 1 kb ladder (Amresco, Solon OH) and 14 lanes of PCR products: lanes 1 & 2, MC1000; lanes 3 & 4, JS81; lanes 5 & 6, JS45; lanes 7 & 8, JS56; lanes 9 & 10, JS87; lanes 11 & 12, JS44; lanes 13 and 14, JS43. Odd numbered lanes show products of PCR1, even numbers lanes those of PCR2

The presence of insertions and/or deletions within the *flhD* promoter in the partially or non-motile MC1000 biofilm isolates was determined with polymerase chain reactions PCR1 and PCR2 (Fig. 2; Table 1, entire PCR1/PCR2 block of columns; [11]). All partially motile isolates, yielded PCR products that were identical in length to those of the MC1000 parent strain. As representative of this group, JS81 is included in Fig. 2b, sequence analysis of its *flhD* operon did not reveal any mutations from the IS5 to the end of the FlhC open reading frame. The non-motile isolates divided into three categories. Category I contained 4 isolates from the 7 day biofilms and 21 isolates from the 14 day biofilms that produced PCR products that were of identical length as those produced by MC1000. As representative of the category I isolates, JS45 is included in Fig. 2b. Sequence analysis revealed that JS45 had a frame shift mutation in codon 96 of FlhC that led to a TGA stop codon in position 97. JS59 and JS68 had a point mutation in codon 67 of FlhC that led to a Trp to Ser change, JS84 had a deletion of 14 base pairs, leading to a frame shift in codon 136 of FlhC and a changed protein sequence thereafter. JS98 had a point mutation in codon 103 of FlhC that converted this codon to a TAG stop codon. Sequence data for these five category I isolates are included in Additional file 2: Figure S1.

Category II contained 6 isolates that failed to produce a PCR product in one of the two reactions. JS56 (Fig. 2b), JS62 and JS79 failed to produce a PCR1 product and produced a PCR2 product that was slightly shorter than that of MC1000. Sequence analyses of the *flhD* operons of these isolates revealed that they had deletions that started at the parental IS5 element and extended to 79 bp (JS62) and 14 bp (JS56 and JS79) downstream of the transcriptional start site of the operon (Additional file 2: Figure S2). For these three isolates, the deleted region included the binding site for the forward primer 1. JS87 is an example of an isolate that did not produce a PCR2 product (Fig. 2b). It produced a PCR1 product of ~ 2.5 kb. Sequence analysis of JS87 revealed that the *flhD* operon contained an IS5 at 139 bp into the open reading frame of FlhD, which is consistent with the length of the PCR1 product. Since we were unable to obtain a PCR2 product, it is likely that there are additional mutations in the area of the forward 2 primer. The other two isolates in this group were JS76 and JS82, both of which produced PCR1 and PCR2 products that were very similar in length to the respective products of JS87.

Category III consisted of 30 isolates that produced PCR products larger than those of MC1000 in both reactions. Of the 30 isolates in this category, 28 isolates produced PCR fragments of 2 and 3.3 kb in PCR1 and PCR2, an example of this group is JS44 (Fig. 2b). This combination of PCR products is indicative of the continued presence of the IS5 from MC1000, supplemented by an insertion of approximately 800 bp within the part of the *flhD* operon that is amplified with the forward 1 primer. The remaining two category III isolates produced PCR1 and PCR2 products that were even larger. These are JS43 (Fig. 2b) and JS70, whose PCR products were 2.6 and 4 kb, respectively.

As examples of category III isolates that produced PCR1 and PCR2 products of 2 and 3.3 kb, we sequenced the *flhD* operons of JS44, JS51, JS58, and JS90 (Additional file 2: Figure S3). JS44 contained an IS1 element in the reverse orientation 54 bp upstream of the ATG start codon of FlhD. On the upstream side, the IS1 was flanked by a 9 bp duplication from the *flhD* promoter sequence. JS51 contained an IS1 element in the forward orientation within the open reading of FlhD at 95 bp downstream of the ATG. JS58 contained an IS1 element in the reverse orientation 5 bp upstream of the ATG start codon of FlhD. This IS1 was flanked downstream by a duplication of the sequence AATAATG, which does not interrupt FlhD’s ATG start codon or its open reading frame. JS90 contained an IS1 element in the forward orientation within the open reading frame for FlhC, 49 bp downstream of the ATG. Sequence analysis with the forward 2 primer revealed the continued presence of the IS5 element from MC1000 in JS44, JS58, and JS90.

**Fig. 3:**
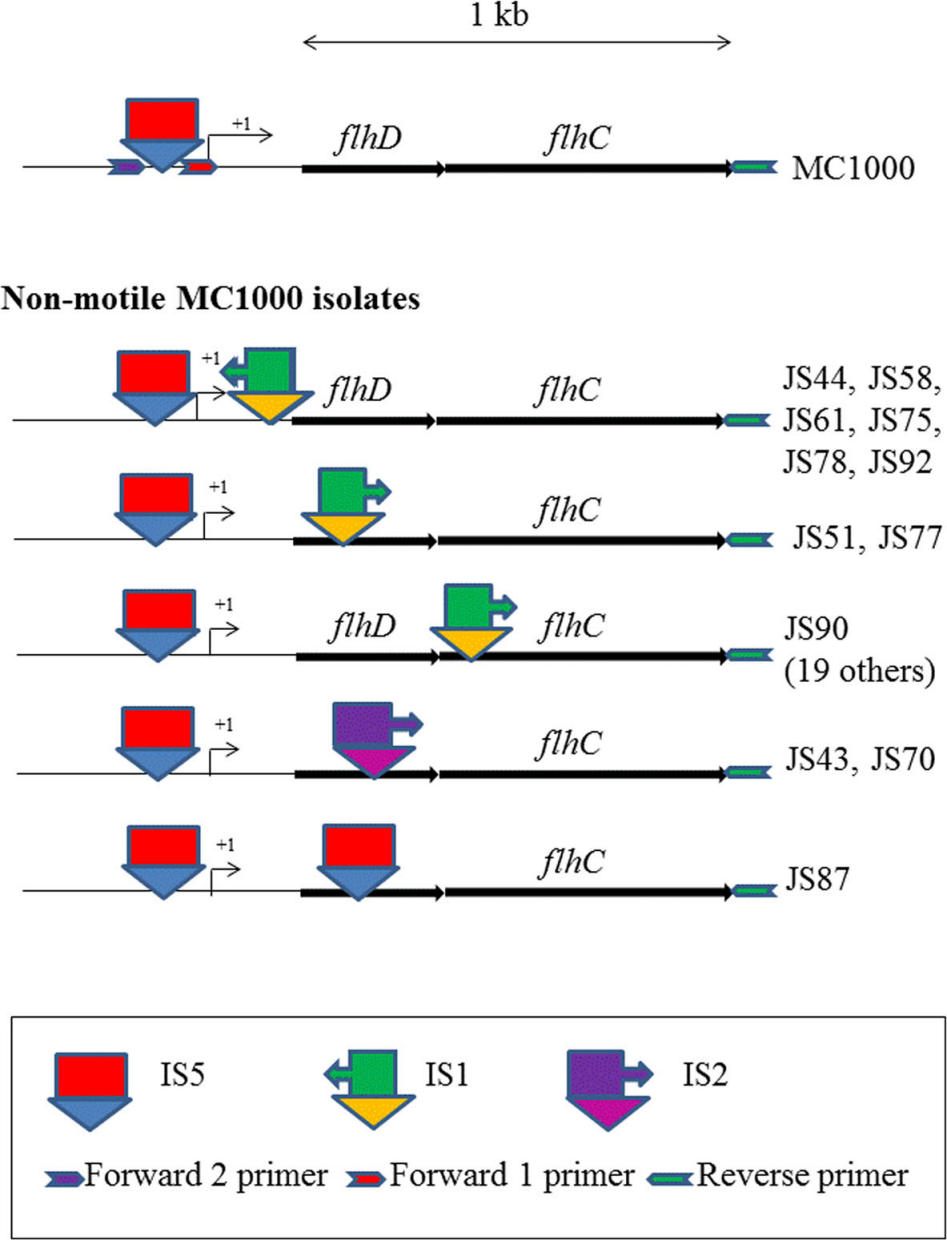
Outcome of the sequence analysis of the category III isolates and PCR3. IS elements and primers are indicated, as are open reading frames. A 1 kb size marker is provided at the top of the Figure. Size is only an approximation. In particular, primers are drawn larger than they really are, while IS elements are drawn smaller

To determine the presence of IS1 elements in the remaining category III isolates, we performed PCR3 (Table 1, PCR3 column, Fig. 3). Among the isolates that had produced PCR1 and PCR2 products of 2 and 3.3 kb in PCR1 and PCR2, we found four more isolates (JS61, JS75, JS78, and JS92) that had IS1 elements in the reverse orientation and at the approximate location as JS44 and JS58. JS77 had the IS1 element inserted in the same orientation at approximately the same location as JS51. An additional 19 isolates had IS1 in the forward orientation and at the approximate location of JS90. These are noted in Additional file 1. Finally, we sequenced the *flhD* operons of the two category III isolates that had produced PCR1 and 2 products larger than 2 and 3.3 kb. JS43 and JS70 had an IS2 element 181 bp into the open reading frame of FlhD. The location of all IS1, IS2, and IS5 elements that were found in the *flhD* operon within this study are included in Fig. 3.

In summary, we identified IS1 elements in the *flhD* operons of 28 of the 62 non-motile isolates that were tested. Two isolates carried an IS2 in their *flhD* gene, one carried an IS5 in *flhD*. Three isolates had deletions in the promoter region of *flhD,* five had small mutations in the open reading frame for FlhC. The IS element insertions were downstream of the transcriptional start of the operon, either between the transcriptional and the translational start of *flhD* or within the open reading frames of FlhD or FlhC. The small mutations were also found in the open reading frame for FlhC. The deletions included the region from the IS5 to the transcriptional start and a portion of the untranslated mRNA transcript.

#### Motility heterogeneity leads to an increase in biofilm biomass after three weeks of incubation

**Fig. 4:**
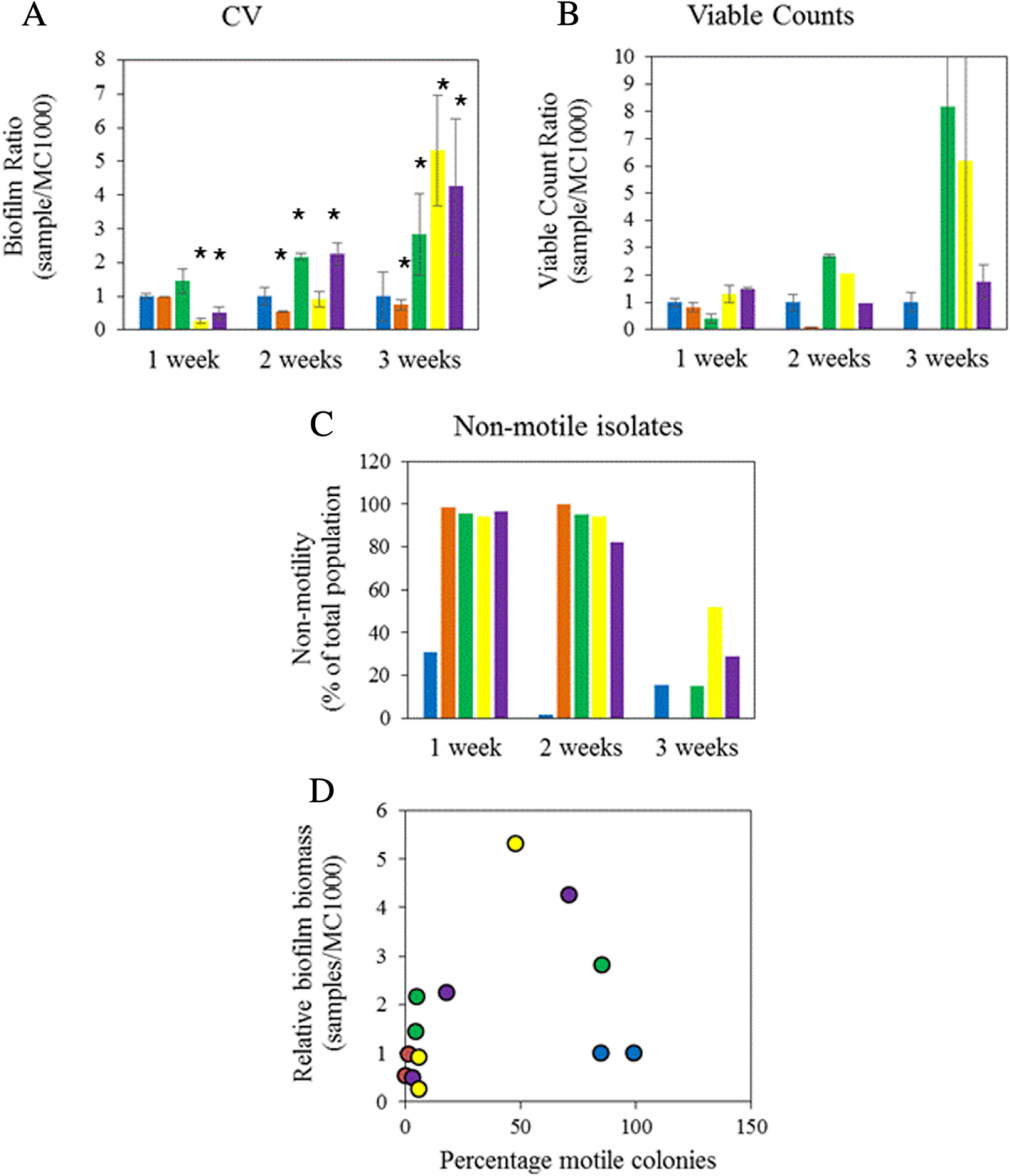
Biofilm biomass in mixed biofilms. Biofilm was grown by MC1000, BP19 and mixtures of MC1000 and BP19 in ratios of 1:10, 1:1, and 10:1. Biofilm amounts were determined weekly for 3 weeks by the CV assay **a**, viable cell counts were determined simultaneously (**b**). **c** indicates the percentage of non-motile bacteria in the biofilm, determined on motility plates. For **a** through **c**, blue bars are used for MC1000, orange bars for BP19, green bars for the 1:1 mixture, yellow for the 10:1 mixture, and purple for the 1:10 mixture. For **d**, data from **a** and **c** were combined and the relative biofilm biomass was plotted versus the percentage of motile isolates. The color code for the circles is identical to the color codes for the bars in (**a**) to (**c**)

Biofilm was incubated for up to 3 weeks, composed of MC1000, BP19 (MC1000 *flhD*::*kn*), or mixtures of MC1000 and BP19 at ratios of 1:1, 10:1, and 1:10 (Fig. 4). Quantification of biofilm with CV after 1 week determined that the 1:1 mixture of MC1000 and BP19 (Fig. 4a, 1 week, green bar) had slightly more biofilm than the pure culture of MC1000 (Fig. 4a, 1 week, blue bar) or BP19 (Fig. 4, 1 week, orange bar) and significantly more biofilm than the remaining two mixtures (Fig. 4a, 1 week, yellow and purple bars). This increase in CV detectable biofilm biomass was not paralleled by an increase in cell viability (Fig. 4b). At this time, non-motile isolates had almost completely taken over the biofilms of the three mixed cultures, whereas MC1000 biofilm contained 15 % non-motile isolates (Fig. 4c). Intriguingly, the biofilm by BP19 contained two motile isolates (out of 400 tested) that were still kanamycin resistant. We can not explain this phenomenon and did not investigate these isolates further.

After 2 weeks, the 1:1 mixture, and the 1:10 mixture contained more biofilm than the other cultures (Fig. 4a). For the 1:1 mixture, this increase in biofilm biomass was paralleled by an increase in viable cell counts (Fig. 4b), for the 1:10 mixture, this was not the case. With the exception of MC1000, biofilms contained primarily non-motile isolates.

After 3 weeks, the three mixed cultures contained up to five times more biofilm than MC1000. For the 1:1 mixture and the 10:1 mixture, this increase was paralleled by an increase in viable cell counts, though admittedly with a large standard error. Biofilm of MC1000 and the three mixed cultures contained a mixture of motile and non-motile isolates. It is noteworthy that biofilm by the non-motile BP19 contained such a small number of viable cells that a CFU count could not be computed (missing bar in Fig. 4b) and motility could not determined (missing bar in Fig. 4c).

To determine whether the non-motile isolates of MC1000 carried mutations similar to the ones earlier described in this study, the non-motile isolates from one week old MC1000 biofilm, designated JS111 to JS125, were further investigated. Transforming the isolates with pXL27 yielded 47 % of motile transformants, indicating that the respective isolate had acquired a mutation in its *flhD* operon. All isolates were subjected to PCR1 and PCR2, the *flhD* operons of JS111and JS119 were sequenced. JS111 had the 1200 bp PCR1 product that is characteristic for MC1000 and a 1400 bp PCR2 product. In agreement with this, sequence analysis revealed the lack of the IS5 element of the MC1000 parental strain. There was a single base pair change from C to A at −63 bp from the transcriptional start. JS119 carried an IS5 at 91 bp before the ATG start codon of FlhD (Additional file 2: Figure S2). As described in Additional file 2: Figure S2 and Fig. 3, we obtained isolates that carried mutations in the *flhD* operon. The nature of the mutations may vary between the experiments.

To visualize the increased biofilm biomass of the heterogeneous biofilm, we combined all data points, regardless of the strain/mixture and the time point (Fig. 4d). For clarification, we maintained the color code from the bar plots (Figs. 4a to c), which means that yellow circles represent the 1:1 mixture of the two strains. Specifically, the yellow 5.5 fold data point at 50 % motility was computed from the yellow bars at 3 weeks in Figs. 4a and c. Throughout all data points, the biofilm that consisted of 50 % motile and 50 % non-motile isolates had by far the largest amount of biomass. In contrast, cultures that consisted of either 100 % motile isolates or 100 % non-motile isolates formed between 5 and 10 fold less biofilm.

## Discussion

This study presents several pieces of evidence to support our hypothesis that i) mutants can arise in the *flhD* operon that decrease motility and that ii) the resulting motility heterogeneity in the biofilm may help the bacteria to maintain biofilm biomass over time.

The first set of experiments led to the conclusion that the biofilm environment may favor a mixture of motile and non-motile bacteria. From biofilms formed by the highly motile MC1000, we recovered 62 non-motile isolates, but no hyper-motile ones. We conclude that moderate selective pressure towards the opposing motility phenotype might operate in this biofilm. If this were true, then the biofilm environment may favor a mixture of motile and non-motile bacteria. To test this hypothesis, we initiated biofilms with MC1000 or with a mixture of MC1000 and the non-motile BP19 and followed the composition of the biofilm for about one month. The biofilm formed by the former degraded, whereas the latter formed a biofilm that maintained its integrity for a month with a mixture of both strains for most of the 1 month time period of the experiment (Fig. 1).

An intriguing phenomenon was observed during this competitive experiment. At 6 h, the *flhD* mutant strain BP19 had completely taken over the biofilm to a percentage that was even on top of 100 % (Fig. 1, second data point of the black squares). While we don’t know why more bacteria had grown on the kanamycin plates than on the plain LB plates, we believe that the massive increase in the *flhD* mutant population might be due to the increase in the cell division rate of the mutants relative to the parent strain that was previously observed [11, 15, 16]. In summary of these previous publications, *flhD* mutants divided to 37 times higher cell densities than isogenic parent bacteria due to a depletion of serine from the bacterial growth media that the *flhD* mutants failed to sense. In agreement with these previous publications, we observe large numbers of *flhD* mutants after 24 h of incubation in all of our experiments with this strain.

Motility heterogeneity has been observed in other environments. One such environment is the mouse intestine. In the streptomycin-treated mouse intestine, *flhD* deletion mutants derived from the originally motile *E. coli* MG1655 parent eventually took over the population, but only after prolonged incubation [17, 18]. Intriguingly, 10 to 20 % of the remaining bacteria had *envZ* missense mutations [19]. EnvZ is the histidine kinase of the osmoregulation system EnvZ/OmpR. The *envZ*_P41L_ mutation increased the levels of phospho-OmpR [20], an inhibitor of *flhD* expression [21]. The authors concluded that the intestine likely contains niches where high *flhD* expression might be the advantage, alongside niches where low *flhD* expression might be an advantage [18]. The researchers did not attribute the entirety of this effect to motility. Instead, they proposed a ‘restaurant’ hypothesis [19], where the many metabolic genes that are regulated by *flhD* [22, 23] contribute to the niche adaptation. Our own studies with the aerotaxis sensor Aer, which impacts the sugar acid degradation pathway, support this hypothesis [24].

A second example of motility heterogeneity can be found in *S. enterica*, where FliZ and YdiV are responsible for the co-existing sub-populations of motile and non-motile bacteria during host colonization. FliZ is a post-translational activator of *flhD* expression [6] and constitutes a positive feed-back loop [25]. The other half of this switch is YdiV, which binds to FlhD/FlhC and inhibits its action upon downstream flagellar genes [26]. FliZ is responsible for the motile sub-population, whereas YdiV partitions cells into the non-motile sub-population [8]. On the basis of our current study, we propose a fundamentally different mechanism by which bacteria can partition their populations into motile and non-motile sub-populations, namely by mutating the *flhD* operon of individual bacteria, modulating FlhD/FlhC expression or producing dys-functional proteins, and thus generating motility heterogeneity within the biofilm.

This study investigates both, the mechanism by which heterogeneity is generated and the long-term impact of such heterogeneity on the biofilm. As mechanism, we propose that the location of the mutation within the *flhD* operon determines whether the effect on motility is positive or negative. All the previously published *E. coli* K-12 biofilm isolates had increased motility [11–14]. Barker and coworkers postulated that the positive effect on *flhD* expression of these insertions may be due to interference with some of the negative regulators (*e.g.* OmpR, RcsB [13, 21, 27–29]) that have binding sites in this region. Many of the non-motile isolates that were recovered from MC1000 in this study carried IS elements in their *flhD* operons that were located either between the transcriptional and the translational start of the operon or within the open reading frames of FlhD or FlhC. This could be attributed to different post-transcriptional causes. For JS58, the IS1 is only 5 bp upstream of the ATG start codon for FlhD. It is more than likely that this would interfere with binding of the ribosome. For JS51 and JS90, the IS1 elements are within the open reading frames of FlhD and FlhC, respectively, an arrangement that has been described for other bacterial species. Within the genus *Shigella*, *Shigella dysenteriae* contains an IS1 in the open reading frame of FlhC, whereas *Shigella flexneri* has an IS1 in the open reading frame of FlhD [30]. Inactivation of FlhC is the obvious reason for the lack of motility in these bacteria.

Other mutations that were found in non-motile isolates of MC1000 biofilm support the conclusions that were drawn from the IS elements. Deletions in the *flhD* operon of JS56, JS62, and JS79 covered the binding site for the forward 1 primer that coincides with the −35 binding site of the RNA polymerase. Likewise, the *flhD* operons of JS45, JS59, JS68, JS84, and JS98 contained point mutations and a 14 bp deletion within the open reading frame for FlhC. These mutations, too, would be expected to render the bacteria non-motile.

The fact that previous studies recovered hyper-motile isolates from *E. coli* biofilm and this study describes the recovery of non-motile isolates from *E. coli* biofilm may appear as a contradiction. However, all previous studies were performed with genetic backgrounds of *E. coli* K-12 that were poorly motile at the beginning of the experiment. In contrast, this study used a highly motile genetic background in MC1000. Together, these observations indicate that there may be a moderate selective pressure towards the respective other motility phenotype in all these experiments. We interpret this as a confirmation of our conclusion that biofilm strives towards a mixture of both motile and non-motile bacteria.

To study the long-term impact of motility heterogeneity on the biofilm, we quantified biofilm biomass from either pure cultures of MC1000, BP19, or mixtures of MC1000 and BP19 (Fig. 4). After 3 weeks, the different mixtures yielded up to 5 times more biofilm biomass than either of the two pure cultures. This was true regardless of the composition of the inoculated mixture. While the viable bacterial counts did not always parallel the results from the CV assay, there is no doubt that the majority of the tested biofilms did indeed contain live bacteria. After 3 weeks, these viable bacteria constituted mixtures of both motile and non-motile bacteria. Again, this was true regardless of the composition of the mixture at time of inoculation. Intriguing observations were made with the *flhD* mutant, BP19. We were unable to perform the experiment for the entirety of the 3 weeks because the biofilm disintegrated. In fact, the 2 weeks biofilm already contained almost no viable bacteria anymore (Fig. 4b). The question remains how the mutant was able to make a biofilm in the first place, considering that flagella are thought to be needed for the initial attachment. It is possible that the bacteria used fimbriae/pili/curli as attachment tools straight from the beginning, as was proposed by A.J. Wolfe’s lab [31] for some of the acetate mutants which were able to produce a fimbriae based biofilm, albeit with a different integrity and smaller amounts of biomass.

Altogether, this experiment leads to the conclusion that motility heterogeneity helps the biofilm to maintain its integrity for a longer period of time. Assuming that a larger biofilm biomass made up primarily of viable bacteria may constitute as an advantage, one could consider this experiment first evidence towards a new hypothesis that motility heterogeneity in a biofilm may constitute a long-term advantage.

## Conclusions

This study attempted to combine investigations of two important mechanisms of adaptation that help bacteria adapt to the heterogenous niches within the biofilm: spontaneous mutations and phenotypic expression. The experiments presented support our hypothesis that mutations can arise in the *flhD* operon of biofilm bound *E. coli* that render the bacteria non-motile. We propose this is a mechanism to generate motility heterogeneity in biofilm and provided evidence that such heterogeneity leads to increases in biofilm biomass after prolonged incubation.

## Methods

### Bacterial strains

MC1000 is a highly motile (5.9 mm/h on motility plates [32]) strain, due to the presence of an IS5 element in its *flhD* promoter [11]. MC1000 forms biofilm poorly [32]. MC1000 *flhD*::*kn*, designated BP19, does not express *flhD*, is non-motile and resistant towards kanamycin [23, 32, 33]. All JS designated isolates were recovered as non-motile or reduced motility colonies from either biofilm or planktonic bacteria of MC1000. Bacterial strains were maintained as freezer stocks at −80 °C and streaked onto Luria Bertani (LB; 1 % tryptone, 0.5 % NaCl, 0.5 % yeast extract) agar plates prior to each experiment.

### Biofilm formation, colony recovery, and motility screening

Biofilms were grown from MC1000 in duplicate, separate wells of a 6 well plate in LB at 32 °C. After 7 and 14 days, liquid growth media was removed and biofilms were washed twice with PBS. Bacteria were suspended in PBS and homogenized by pipetting them up and down the pipette tip several times. The homogenates were serially diluted, and plated on LB agar plates. As a control experiment, we grew bacteria in bottles under shaking and serially diluted the planktonic bacteria in the growth medium. Within each experiment, we picked several hundreds of isolated colonies (referred to as isolates) from the plates and screened them for motility. This was done on semi solid agar plates made from tryptone broth (TB; 1 % tryptone, 0.5 % NaCl) and 0.3 % agar [34]. Plates were incubated for 4–8 h at 32 °C in a humid environment. The diameters of the swarm rings were compared between the parent strain and the respective derivatives. Motility phenotypes were confirmed by testing the respective isolate on motility plates two more times. Isolates that reproducibly produced a motility phenotype that was different from their parent were maintained as freezer stocks and given a JS designation. Note that colony designations take the different strains and time points under consideration, but do not distinguish between the two replicate experiments.

### Long-term biofilm experiments

The first long-term experiment followed the protocol described for the recovery of the isolates, but expanded the incubation time to 4 weeks. Since the biofilm disintegrated during this time, we then inoculated the cultures with a 1:1 starting mixture of MC1000 and its isogenic MC1000 *flhD*::*kn* mutant. This was done in LB on multiple 35 mm petri dishes made of polystyrene, which were incubated at 32 °C. Biofilms were grown over the course of 28 days. At each time point, individual petri dishes were removed from the incubator and processed. Bacterial growth medium was removed, biofilms were washed twice with PBS, bacteria were serially diluted, and plated onto LB agar plates and LB plates, supplemented with 50 μg/ml kanamycin. The total number of bacteria was presented as colony forming units per well (CFU/well) and was calculated from the LB plates. To determine the percentage of *flhD* mutants, bacterial counts from the kanamycin plates were divided by total bacterial numbers. The experiment was done twice in two replicates each, averages and standard deviations were calculated across the four experiments.

### Molecular characterization of the non-motile isolates from MC1000 biofilm

To test whether some of the non-motile MC1000 biofilm isolates might contain mutations within their *flhD* operons in addition to the IS5, all 85 isolates were transformed with the *flhD* and *flhC* expressing plasmid pXL27 [35] and tested for complementation of the motility phenotype. Plasmid pXL27 was moved into the respective bacterial isolates via chemical transformation, taking advantage of the penicillin resistance gene on the plasmid as a selective marker. For each non-motile colony, 8 independent transformants were tested on motility plates as described [34].

To detect insertions and deletions within the *flhD* operon, two PCR reactions (PCR1 and PCR2) were performed that were originally designed to detect insertions of IS elements within the *flhD* promoter [11]. The PCR 1 fragment (Fig. 2a) starts downstream of the published [11, 12] hot spot for IS1 and IS5 in the *flhD* promoter and ends at the 3’-end of the *flhC* open reading frame. This fragment is expected to be 1199 bp in length in the absence of insertions. The PCR2 fragment (Fig. 2a) starts upstream of the hotspot. PCR2 is expected to yield a 1343 bp fragment in the absence of IS elements. PCR1 was done with forward primer 1, PCR2 with forward primer 2. Both reactions used the same reverse primer. Note that the sequences for these and all other PCR and sequencing primers are listed in Additional file 2: Table S1.

For the MC1000 parent strain, the expectation was a PCR2 fragment of 2538 bp together with a PCR1 fragment of 1199 bp length. This is due to the IS5 between the binding sites of the forward primers 1 and 2. For MC1000 isolates that produced PCR1 and PCR2 fragments of those same lengths, this would indicate that there are not any large deletions or insertions besides the parental IS5 (category I isolates). The lack of a PCR1 or PCR2 product might point towards mutations in the area of the respective primer binding site (category II isolates). Should both PCR fragments be longer than 1343 bp and 1199 bp, this would be indicative of an insertion downstream of the forward primer 1 (category III isolates).

As one example of the partially motile isolates, we sequenced the *flhD* operon of JS81 and compared it to that of MC1000. As representatives of the category I isolates that produced PCR1 and PCR2 products of parental length, we sequenced the *flhD* operons of JS45, JS59, JS68, JS84, and JS98. As examples of the category II isolates that did not produce a PCR1 product, we sequenced the *flhD* operons of JS56, JS62 and JS79, as one example of a category II isolate that did not produce a PCR2 product, we sequenced the *flhD* operon of JS87. Among the category III isolates that produced PCR1 and PCR2 products of 2 kb and 3.3 kb respectively, the *flhD* operons from JS44, JS51, JS58, JS78, and JS90 were sequenced. Also sequenced were the *flhD* operons of JS43 and JS70, the category III isolates that produced PCR1 and PCR2 products longer than 2 and 3.3 kb. Sequence analysis was done from PCR product with the Sanger technique by Macrogen (Rockville, MD). Primers that were used to produce the PCR product, as well as primers that were used for the sequencing reactions are listed in Additional file 2: Table S2.

PCR3 was performed to test for the presence, approximate location, and orientation of IS1 elements in the remaining isolates that had produced larger PCR1 and PCR2 products than their parent MC1000 strain. We used forward primer 3A and 3B together with the reverse primer to detect the two different possible orientations of the IS1. The presence of a PCR product by forward primer 3A is indicative of IS1 in forward orientation. A PCR product yielded by forward primer 3B is indicative of IS1 in reverse orientation. The length of the respective PCR fragment is indicative of the distance from the binding site for the reverse primer.

### Determination of the effect of motility heterogeneity on biofilm biomass after prolonged incubation

We inoculated 6 well polystyrene plates with MC1000, BP19, and mixtures of MC1000 and BP19 at ratios of 1:1, 10 MC1000/1 BP19 (designated 10:1), and 1 MC1000/10 BP19 (designated 1:10) in TB. Plates were covered with adhesive film (VWR International, Chicago IL) and incubated at 34 °C. Biofilm amounts were determined after 1, 2, and 3 weeks with the crystal violet assay (CV) [36]. Briefly, the planktonic bacteria were removed and the biofilms were washed twice with PBS, dried for 10 min, and stained for 10 min with 0.1 % crystal violet in ddH_2_O. The CV solution was removed and the biofilms were washed twice with PBS. CV was extracted from the bacteria with a mixture of 80 % ethanol and 20 % acetone, diluted 1:10, and the OD_600_ was determined with a Synergy H1 plate reader from Biotek Instruments (Winooski, VT). Relative biofilm biomass is expressed as ratios where the OD_600_ of each of the cultures was divided by that of MC1000. Averages and standard deviations were calculated across the three replicates for each strain/mixture. A Student’s *t*-test was used to determine the statistical significance of the difference between each mixture (or the non-motile mutant) and the MC1000 parent strain. A *p*-value below 0.05 was considered significant.

On separate 6 well plates from the above experiment, biofilms were re-suspended, serially diluted, and bacteria were isolated by spreading the dilutions onto LB agar plates. Viability counts were calculated from these plates and expressed as ratios as described for the CV ratio. For the motility test, at least 100 isolated colonies per culture and time point were spotted on motility plates. An exception from this was BP19, whose biofilm did not permit the recovery of 100 colonies after 3 weeks of incubation. Motility was expressed as percentage of non-motile isolates within the total population, calculated across all isolates for the respective strain/mixture and time point. In a separate analysis, all data points were combined, regardless of strain/mixture or time point. Each data point was plotted as relative biofilm biomass from the CV assay versus the percentage of motile isolates.

Non-motile colonies from 1 week old MC1000 biofilm were designated JS111 through JS125 and kept at −80 °C. These isolates were transformed with pXL27 and tested for complementation of the motility phenotype. PCR1 and PCR2 were performed with all these isolates, the *flhD* operons of JS111 and JS119 were sequenced.

## Additional files

Additional file 1: Complete data set for individual isolates. (XLS 41 kb)
Additional file 2: Sequence analysis and PCR primers. (DOCX 75 kb)

## Abbreviation

CV: Crystal violet

## References

[1] Murata M, Fujimoto H, Nishimura K, Charoensuk K, Nagamitsu H, Raina S, Kosaka T, Oshima T, Ogasawara N, Yamada M (2011). Molecular strategy for survival at a critical high temperature in *Escherichia coli*. PLoS One.

[2] White-Ziegler CA, Um S, Perez NM, Berns AL, Malhowski AJ, Young S (2008). Low temperature (23° C) increases expression of biofilm-, cold-shock- and RpoS-dependent genes in *Escherichia coli* K-12. Microbiology.

[3] Chang HH, Cohen T, Grad YH, Hanage WP, O’Brien TF, Lipsitch M (2015). Origin and proliferation of multiple-drug resistance in bacterial pathogens. Microbiol Mol Biol Rev.

[4] Vlamakis H, Aguilar C, Losick R, Kolter R (2008). Control of cell fate by the formation of an architecturally complex bacterial community. Genes Dev.

[5] Samanta P, Clark ER, Knutson K, Horne SM, Prüß BM (2013). OmpR and RcsB abolish temporal and spatial changes in expression of *flhD i*n *Escherichia coli* biofilm. BMC Microbiol.

[6] Saini S, Brown JD, Aldridge PD, Rao CV (2008). FliZ Is a posttranslational activator of FlhD_4_C_2_-dependent flagellar gene expression. J Bacteriol.

[7] Stewart MK, Cookson BT (2012). Non-genetic diversity shapes infectious capacity and host resistance. Trends Microbiol.

[8] Stewart MK, Cookson BT (2014). Mutually repressing repressor functions and multi-layered cellular heterogeneity regulate the bistable *Salmonella fliC* census. Mol Microbiol.

[9] Relman DA (2011). Microbial genomics and infectious diseases. N Engl J Med.

[10] Zhang Z, Saier MH (2011). Transposon-mediated adaptive and directed mutations and their potential evolutionary benefits. J Mol Microbiol Biotechnol.

[11] Barker CS, Prüß BM, Matsumura P (2004). Increased motility of *Escherichia coli* by insertion sequence element integration into the regulatory region of the *flhD* operon. J Bacteriol.

[12] Wang X, Wood TK (2011). IS5 inserts upstream of the master motility operon *flhDC* in a quasi-Lamarckian way. ISME J.

[13] Lee C, Park C (2013). Mutations upregulating the *flhDC* operon of *Escherichia coli* K-12. J Microbiol.

[14] Fahrner KA, Berg HC (2015). Mutations that stimulate *flhDC* expression in *Escherichia coli* K-12. J Bacteriol.

[15] Prüß BM, Matsumura P (1996). A regulator of the flagellar regulon of *Escherichia coli*, *flhD*, also affects cell division. J Bacteriol.

[16] Prüß BM, Markovic D, Matsumura P (1997). The *Escherichia coli* flagellar transcriptional activator *flhD* regulates cell division through induction of the acid response gene *cadA*. J Bacteriol.

[17] Gauger EJ, Leatham MP, Mercado-Lubo R, Laux DC, Conway T, Cohen PS (2007). The role of motility and the *flhDC* operon in *Escherichia coli* MG1655 colonization of the mouse intestine. Infect Immun.

[18] Leatham MP, Stevenson SJ, Gauger EJ, Krogfelt KA, Lins JJ, Haddock TL, Autieri SM, Conway T, Cohen PS (2005). Mouse intestine selects non-motile *flhDC* mutants of *Escherichia coli* MG1655 with increased colonizing ability and better utilization of carbon sources. Infect Immun.

[19] Leatham-Jensen MP, Frimodt-Moller J, Adediran J, Mokszycki ME, Banner ME, Caughron JE, Krogfelt KA, Conway T, Cohen PS (2012). The streptomycin-treated mouse intestine selects *Escherichia coli envZ* missense mutants that interact with dense and diverse intestinal microbiota. Infect Immun.

[20] Adediran J, Leatham-Jensen MP, Mokszycki ME, Frimodt-Moller J, Krogfelt KA, Kazmierczak K, Kenney LJ, Conway T, Cohen PS (2014). An *Escherichia coli* Nissle 1917 missense mutant colonizes the streptomycin-treated mouse intestine better than the wild type but is not a better probiotic. Infect Immun.

[21] Shin S, Park C (1995). Modulation of flagellar expression in *Escherichia coli* by acetyl phosphate and the osmoregulator OmpR. J Bacteriol.

[22] Prüß BM, Liu X, Hendrickson W, Matsumura P (2001). FlhD/FlhC-regulated promoters analyzed by gene array and *lacZ* gene fusions. FEMS Microbiol Lett.

[23] Prüß BM, Campbell JW, Van Dyk TK, Zhu C, Kogan Y, Matsumura P (2003). FlhD/FlhC is a regulator of anaerobic respiration and the Entner-Doudoroff pathway through induction of the methyl-accepting chemotaxis protein Aer. J Bacteriol.

[24] Horne SM, Mattson KR, Prüß BM. An *Escherichia coli aer* mutant exhibits a reduced ability to colonize the streptomycin-treated mouse large intestine. Ant van Leeuwenhoek Internat J Gen Mol Microbiol. 2009;95:149–158. 10.1007/s10482-008-9298-z 19130287

[25] Saini S, Koirala S, Floess E, Mears PJ, Chemla YR, Golding I, Aldridge C, Aldridge PD, Rao CV (2010). FliZ induces a kinetic switch in flagellar gene expression. J Bacteriol.

[26] Wada T, Hatamoto Y, Kutsukake K (2012). Functional and expressional analyses of the anti-FlhD_4_C_2_ factor gene *ydiV* in *Escherichia coli*. Microbiology.

[27] Hagiwara D, Sugiura M, Oshima T, Mori H, Aiba H, Yamashino T, Mizuno T (2003). Genome-wide analyses revealing a signaling network of the RcsC-YojN-RcsB phosphorelay system in *Escherichia coli*. J Bacteriol.

[28] Lehnen D, Blumer C, Polen T, Wackwitz B, Wendisch VF, Unden G (2002). LrhA as a new transcriptional key regulator of flagella, motility and chemotaxis genes in *Escherichia coli*. Mol Microbiol.

[29] Ko M, Park C (2000). H-NS dependent regulation of flagellar synthesis is mediated by a LysR family protein. J Bacteriol.

[30] Al Mamun AA, Tominaga A, Enomoto M (1996). Detection and characterization of the flagellar master operon in the four *Shigella* subgroups. J Bacteriol.

[31] Wolfe AJ, Chang DE, Walker JD, Seitz-Partridge JE, Vidaurri MD, Lange CF, Prüß BM, Henk MC, Larkin JC, Conway T (2003). Evidence that acetyl phosphate functions as a global signal during biofilm development. Mol Microbiol.

[32] Prüß BM, Verma K, Samanta P, Sule P, Kumar S, Wu J, Horne SM, Christianson DA, Stafslien SJ, Wolfe AJ, Denton A (2010). Environmental and genetic factors that contribute to *Escherichia coli* K-12 biofilm formation. Arch Microbiol.

[33] Malakooti J. Molecular analysis of the *Escherichia coli* motor components and regulation of internal control elements of the *flaA* operon. Dissertation, University of Illinois at Chicago; 1989.

[34] Wolfe AJ, Berg HC (1989). Migration of bacteria in semisolid agar. Proc Natl Acad Sci U S A.

[35] Liu X, Matsumura P (1994). The FlhD/FlhC complex, a transcriptional activator of the *Escherichia coli* flagellar class II operons. J Bacteriol.

[36] O’Toole GA, Pratt LA, Watnick PI, Newman DK, Weaver VB, Kolter R (1999). Genetic approaches to study of biofilms. Methods Enzymol.

