# Supplementary material for: Spontaneous mutations in the flhD operon generate motility heterogeneity in Escherichia coli biofilm
Source: BMC Microbiol. 2016 Nov 8;16:262. doi: 10.1186/s12866-016-0878-1 (PMC5100188; doi:10.1186/s12866-016-0878-1)
Supplement: Additional file 2: — Sequence analysis and PCR primers. (DOCX 75 kb) [file 12866_2016_878_MOESM2_ESM.docx]

**Supplemental Figure 1:**

Sequence analysis of the FlhC open reading frames of the category I isolates that produced PCR1 and PCR2 products whose length was identical or very close to the respective PCR products of the MC1000 parent. The base pairs that are highlighted in red are changed in the indicated isolate. The change is indicated above the DNA sequence. Base pairs highlighted in cyan or yellow indicate the frame shifted new codons in the mutant. The corresponding changes in the amino acids are indicated below the DNA sequence.

Codon

1. **ATG AGT GAA AAA AGC ATT GTT CAG GAA GCG CGG GAT ATT CAG CTG GCA**
2. **ATG GAA TTG ATC ACC CTG GGC GCT CGT TTG CAG ATG CTG GAA AGC GAA**
3. **ACA CAG TTA AGT CGC GGA CGC CTG ATA AAA CTT TAT AAA GAA CTG CGC**
4. **GGA AGC CCA CCG CCG AAA GGC ATG CTG CCA TTC TCA ACC GAC TGG TTT**

C in JS59 and JS68

1. **ATG ACC TGG GAA CAA AAC GTT CAT GCT TCG ATG TTC TGT AAT GCA TGG**

Missing in JS45

TRP

SER

1. **CAG TTT TTA CTG AAA ACC GGT TTG TGT AAT GGC GTC GAT GCG GTG ATC**

ALA

ALA

G in JS98

VAL

Stop

1. **AAA GCC TAC CGT TTA TAC CTT GAA CAG TGC CCA CAA GCA GAA GAA GGA**

TYR

Stop

1. **CC ACTG CTG GCA TTA ACC CGT GCC TGG ACA TTG GTG CGG TTT GTT GAA**

Missing in JS84

1. **AGT GGA TTA CTG CAA CTT TCC AGC TGC AAC TGC TGC GGC GGC AAT TTT**

SER

LEU

LEU

LEU

1. **ATT ACC CAC GCT CAC CAG CCT GTT GGC AGC TTT GCC TGC AGC TTA TGT**
2. **CAA CCG CCA TCC CGG GCA GTA AAA AGA CGT AAA CTT TCC CAG AAT CCT**
3. **GCC GAT ATT ATC CCA CAA CTG CTG GAT GAA CAG AGA GTA CAG GCT GTT**

**225 TAA**

**Supplemental Figure 2:**

Sequence analysis of the *flhD* operons of the category II isolates that failed to produce a PCR1 product. The portion of the sequence that is highlighted in light grey is representative of the deletion in JS62. Deletions in JS56 and JS79 also start at the IS5 element and include both, the light grey and dark grey highlighted portions of the sequence.

**Supplemental Figure 3:**

Sequence analysis of the *flhD* operons of category III isolates. Open reading frames for FlhD and FlhC are marked in bold, start and stop codons are underlined. The horizontal arrow marks the transcriptional start. Sequences highlighted in grey indicate the binding sites for the forward 2, forward 1, and reverse primer (in order). The backbone of the sequence resembles any E. coli K-12 strain that does not carry an IS element in the *flhD* promoter, insertion sites of IS elements are highlighted for IS5 from MC1000 (TTAA) and the IS elements from the derivative isolates. Vertical arrows mark the precise insertion sites, green arrows over green highlighted text indicate a positive effect on *flhD e*xpression, red arrows over red highlighted text a negative effect. Solid lines above the sequence indicate the two binding sites for phosphorylated OmpR [21]. The dashed line above the sequence marks the binding site for RcsAB [Wehland and Bernhardt, JBC, 2000].

1 AGTTATTTTGAC TGTGCGCAAC ATCCCATTTC GATTATTCCT GTTTCATTTT TGCTTGCT

ISI (JS1)

ISI (JS11)

61 AGCGTAGCGAAA AACTTTTTAA CAGATTGAAA TACACCCAA AACAAAAGTA TGACTTATA

IS5 (MC1000)

121 CATTTATGTTA AGTAATTGAG TGTTTTGTGT GATCTGCAT CACGCATTAT TGAAAATCGCA

+1

181 GCCCCCCTCC GTTGTATGTG CGTGTAGTGA CGAGTACAGT TGCGTCGATT TAGGAAAAAT

241 CTTAGATAAG TGTAAAGACC CATTTCTATT TGTAAGGACA TATTAAACCA AAAAGGTGGT

301 TCTGCTTATT GCAGCTTATC GCAACTATTC TAATGCTAAT TATTTTTTAC CGGGGCTTCC

IS1 (JS44)

IS1 (JS58)

361 CGGCGACATC ACGGGGTGCG GTGAAACCGC ATAAAAATAA AGTTGGTTAT TCTGGGTGGG

ISI (JS78)

**FlhD**

421 AATA**ATGCAT ACCTCCGAGT TGCTGAAACA CATTTATGAC ATCAACTTGT CATATTTACT**

IS1 (JS51)

481 **ACTTGCACAG CGTTTGATTG TTCAGGACAA AGCGTCCGCT ATGTTTCGTC TCGGCATAAA**

IS5 (JS87)

541 **TGAAGAAATG GCGACAACGT TAGCGGCACT GACTCTTCCG CAAATGGTTA AGCTGGCAGA**

IS2 (JS43 an JS70)

601 **AACCAATCAA CTGGTTTGTC ACTTCCGTTT TGACAGCCAC CAGACGATTA CTCAGTTGAC**

661  **GCAAGATTCC CGCGTTGACG ATCTCCAGCA AATTCATACC GGCATCATGC TCTCAACACG**

**FlhC**

721 **CTTGCTGAAT GATGTTAATC AGCCTGAAGA AGCGCTGCGC AAGAAAAGGG CCTGA**TC**ATG**

IS1 (JS90)

781 **AGTGAAAAAA GCATTGTTCA GGAAGCGCGG GATATTCAGC TGGCAATGGA ATTGATCACC**

841 **CTGGGCGCTC GTTTGCAGAT GCTGGAAAGC GAAACACAGT TAAGTCGCGG ACGCCTGATA**

901 **AAACTTTATA AAGAACTGCG CGGAAGCCCA CCGCCGAAAG GCATGCTGCC ATTCTCAACC**

961 **GACTGGTTTA TGACCTGGGA ACAAAACGTT CATGCTTCGA TGTTCTGTAA TGCATGGCAG**

1021 **TTTTTACTGA AAACCGGTTT GTGTAATGGC GTCGATGCGG TGATCAAAGC CTACCGTTTA**

1081 **TACCTTGAAC AGTGCCCACA AGCAGAAGAA GGACCACTGC TGGCATTAAC CCGTGCCTGG**

1141 **ACATTGGTGC GGTTTGTTGA AAGTGGATTA CTGCAACTTT CCAGCTGCAA CTGCTGCGGC**

1201 **GGCAATTTTA TTACCCACGC TCACCAGCCT GTTGGCAGCT TTGCCTGCAG CTTATGTCAA**

1261 **CCGCCATCCC GGGCAGTAAA AAGACGTAAA CTTTCCCAGA ATCCTGCCGA TATTATCCCA**

1321 **CAACTGCTGG ATGAACAGAG AGTACAGGCT GTTTAACTGATACGGTGAGGCGCAACATTCC**

**Supplemental Table 1:** Sequences of primers used for PCR and sequencing reactions

| **Primer name** | **Sequence** | **Purpose** |
| --- | --- | --- |
| (Barker) Forward 1 | 5’-CCCCCTCCGTTGTATGTGCG-3’ | For PCR1 [11] |
| (Barker) Forward 2 | 5’-CCTGTTTCATTTTTGCTTGCTAGC-3’ | For PCR2 [11] |
| Forward 3A | 5’-TATGAGCCTGCTGTCACCCTTTGA-3’ | For PCR3: Indicative of an IS1 element in the reverse orientation |
| Forward 3B | 5’-TTCAGGTTATGCCGCTCAATTCGC-3’ | For PCR3: Indicative of an IS1 element in the forward orientation0 |
| Lee forward 2 | 5’-GTGTAACCGCAACAGCGACA-3’ | Production of PCR products for sequence analysis [13] |
| IS5 forward | 5’-ACAGCATCCACGCAAGAA-3’ | Sequence analysis out of IS5 towards *flhD* |
| IS5 reverse | 5’-ATGGTTTCCAGCGGATAAGG-3’ | Sequence analysis out of IS5 away from *flhD* |
| (Barker) Reverse | 5’-GGAATGTTGCGCCTCACCG-3’ | For PCR1/2/3 and sequence analysis [11] |
| *flhD* region forward | 5’-TGGCAGGAGTGCTTCATTAG-3’ | Amplify PCR products for sequencing |
| *flhD* region reverse | 5’-CAGAGCCAGCAGATCCATATAC-3’ | Amplify PCR products for sequencing |

**Supplemental Table 2:** Primers used for sequence analysis

| **Category** | **JS Isolate** | **PCR forward primer** | **PCR reverse primer** | **Sequencing primer** |
| --- | --- | --- | --- | --- |
| PM | JS81 | Forward 2 | Reverse | IS5 forward, Reverse |
| Cat. I | JS45 | Forward 2 | Reverse | IS5 forward, Reverse |
|  | JS59 | Forward 2 | Reverse | IS5 forward, Reverse |
|  | JS68 | Forward 2 | Reverse | IS5 forward, Reverse |
|  | JS84 | Forward 2 | Reverse | IS5 forward, Reverse |
|  | JS98 | Forward 2 | Reverse | IS5 forward, Reverse |
| Cat. II, no PCR 1 product | JS62 | Forward 2 | Reverse | IS5 forward |
|  | JS56 | Forward 2 | Reverse | IS5 forward |
|  | JS79 | Forward 2 | Reverse | IS5 forward |
| Cat. II, no PCR 2 product | JS76 | Forward 1 | Reverse | ND |
|  | JS82 | Forward 1 | Reverse | ND |
|  | JS87 | Forward 1 | Reverse | Forward 1, Reverse, IS5 reverse |
| Cat. III, 2 and 3.3 kb in PCR1 and PCR2 | JS44 | Forward 2 | Reverse | Forward 1 and 2, reverse primers |
|  | JS51 | Forward 1 | Reverse | Forward 1 |
|  | JS58 | Forward 2 | Reverse | Forward 1 and 2, reverse primers |
|  | JS90 | Forward 2 | Reverse | Forward 1 and 2, reverse primers |
| Cat. III, large PCR fragments | JS43 | *flhD* region forward | *flhD* region reverse | Forward 1 |
|  | JS70 | *flhD* region forward | *flhD* region reverse | Forward 1 |
|  | JS78 | Forward 2 | Reverse | IS5 forward |
| NM MC1000, mixed | JS111 | *flhD* region forward | *flhD* region reverse | Forward 2 |
|  | JS119 | Forward 1 | Reverse | Forward 1, reverse |
